# Use of Zoledronic Acid in Paediatric Craniofacial Fibrous Dysplasia

**DOI:** 10.1155/2016/2329483

**Published:** 2016-09-22

**Authors:** Chiara Di Pede, Sabrina Congedi, Sara Rossin, Antuan Divisic, Alesandra De Gregorio, Caterina Agosto, Igor Catalano, Alessandro Mazza, Leonardo Sartori, Stefano Masiero, Franca Benini

**Affiliations:** ^1^Department of Women's and Children's Health, University of Padua, 3 Giustiniani Street, 35128 Padua, Italy; ^2^Pediatric Pain and Palliative Care Service, University of Padua, 59 Ospedale Civile Street, 35121 Padua, Italy; ^3^Department of Medical and Surgical Sciences, University of Padua, Via Giustiniani 2, 35128 Padua, Italy; ^4^Rehabilitation Section, Department of Neuroscience, University of Padua, Via Giustiniani 2, 35128 Padua, Italy

## Abstract

We describe a case of a paediatric patient affected by mandibular fibrous dysplasia (FD) with severe and chronic pain who was successfully treated with zoledronic acid (ZOL): a third-generation bisphosphonate. Further research is needed to assess its safety and efficacy as a treatment option for FD in the paediatric population.

## 1. Introduction 

Fibrous dysplasia (FD) is a benign fibroosseous bone disease characterized by the replacement of bone with cellular fibrous tissue [1]. It is a genetic condition caused by a postzygotic, activating mutation of GNAS (on chromosome 20), a gene encoding the *α*-subunit of the stimulatory G-protein; in mutated cells, this alteration results in a constitutive activation of adenylate cyclase and in a high production of cAMP affecting the proliferation and differentiation of preosteoblasts which leads to overproduction of fibrotic bone matrix [1].

In over 80% of cases, FD affects only one bone site (monostotic) but two or more bone sites can be involved (polyostotic, PFD). Skin lesions, known as Cafè-au-lait macules and endocrinopathies, are typical of the polyostotic form, called McCune-Albright Syndrome. When the skull is affected, the disease is called craniofacial FD [2]. Malignant transformations are rare and most often happen in the third or fourth decade of life [3]. Moreover, FD can cause bone deformities, bone pain, fractures, and nerve compression. In patients affected by FD, bone scan and serum and urine bone reabsorptions markers are relatively increased.

Pain is common in FD and is often the presenting symptom of the disease [1, 2]. Pathophysiology of bone pain in FD remains uncertain but we know for sure that adequate pain management is clearly required to maintain a good functional status and quality of life in these patients.

Analgesic drugs most commonly used to treat FD pain are nonsteroidal anti-inflammatory drugs, opiates, and bisphosphonates.

Recently, encouraging results support the use of bisphosphonates drugs (BD) to treat FD in adults; BD inhibit excessive osteoclastic bone resorption. These drugs reduce not only the biochemical markers of bone turnover but also bone pain and refill the radiographic sites of osteolytic activity. However, the responses to treatment are heterogeneous, as well as the effects on pathological lesions evaluated with X-ray images.

Zoledronic acid is a third-generation bisphosphonate and few data are available regarding its use as a treatment in craniofacial FD; furthermore, experiences concerning the use of bisphosphonates in paediatric age are very limited.

Here, we report our clinical experience in the use of zoledronic acid for intractable pain in an adolescent male with craniofacial FD; we will also review the available literature on the use of bisphosphonates in paediatric population with FD.

## 2. Case Presentation 

A 12-year-old boy was referred to our service for an antalgic evaluation in May 2014 with a diagnosis of fibrous dysplasia of the right hemimandible, which was made at another institution after an incisional biopsy in April 2013.

The mandibular CT showed an irregular, enlarged, and blown aspect of the horizontal branch of the right hemimandible. Body scanning using plain radiographs and cranial CT was performed and led to exclusion of a polyostotic form. A surgical approach was excluded because of the young age.

In the last year, the patient reported a severe and persistent right hemimandibular pain, interfering with sleep and the activities of daily living; no response to acetaminophen was reported; a partial and transient reduction of pain was obtained with ketoprofen and prednisone.

On admission, he was suffering and his pain intensity was 6/10 on NRS scale.

He reported a 6/10 daily pain with peaks of 8/10.

The patient described the pain as pulsating, pressing, and burning, which is so frequent and profound that it interfered with daily activities and sleep. Pain intensity was exacerbated by chewing and was alleviated by playing with friends. The patient referred embarrassment for his weight gain and for his moon-face, caused by corticosteroid therapy. He reported no speech or swallowing problems.

**Figure 1:**
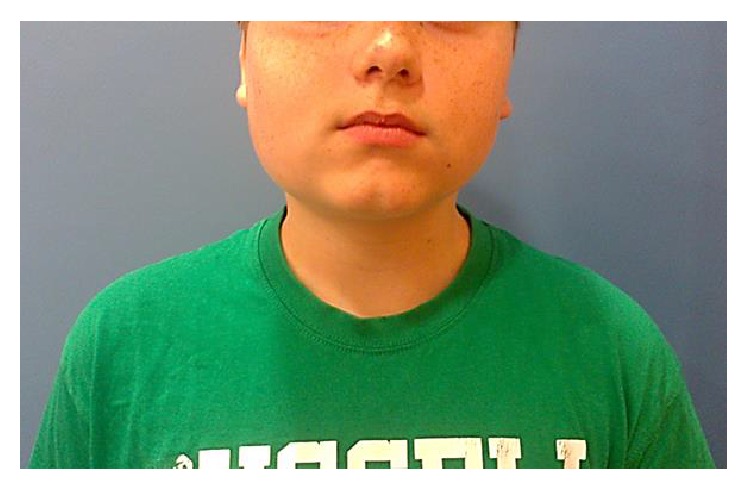
Evident asymmetry of the face.

**Figure 2:**
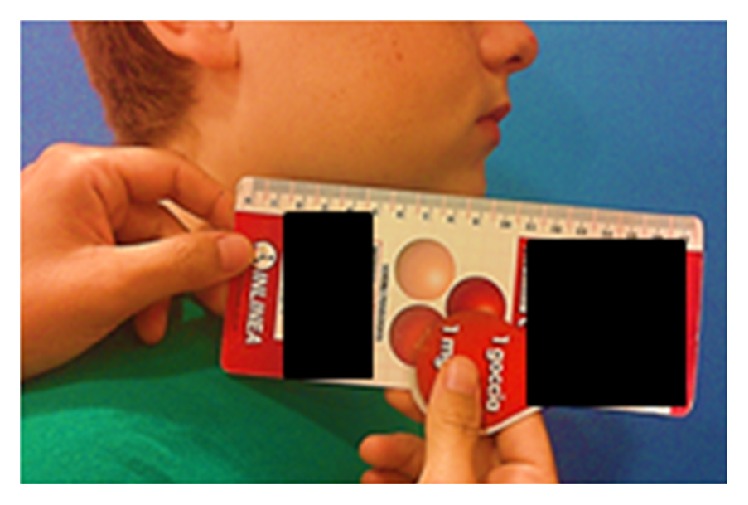
Tumefaction of right mandible corner.

The pain interfered with school activity: sometimes the patient's parents had to bring him the medicines at school, the patient could not attend his lessons, and the pain could get so strong that he could not do his homework. This situation caused a great and deep mutation in family structure: the mother changed her job to have more time to spend with her son and the father changed his work timetable in order to spend more time at home. The pain and its management caused a downturn in the mood of the whole family and a reduction of leisure activities. The patient's parents were afraid of a possible drug addiction. Indeed, the patient had a great anticipatory anxiety regarding the pain and controlled it through an autonomous assumption of drugs. Cranial examination revealed a facial asymmetry, characterized by a swelling of the right cheek (Figures 1 and 2). On palpation, a tender tumefaction on the right mandibular corner was present. Tactile, pain, and thermal sensibilities were preserved. No hyperalgesia, allodynia, nor pain irradiation were present. Oral cavity inspection did not reveal alterations. The mandibular range of motion (ROM) was preserved. Chewer muscles strength was good. No pain was present to acupressure of the temporomandibular joint. No clicks or crepitus was listened during movements of the joint. Cervical rachis ROM was preserved. His weight was 60 kg (+10 kg in the last year).

When the boy was admitted to our service, his drug therapy was as follows: prednisone (12,5 mg/day, p.o.) and acetylsalicylic acid (100 mg 1-2/day, p.o.). This treatment was associated with partial and transient benefit.

On the basis of clinical history, physical examination, and pain characteristics, we diagnosed a nociceptive persistent pain and a multidisciplinary approach was proposed, both pharmacological and psychological. Thus, we suggested to complete a pain diary, in which the patient and/or his parents could record specific data about pain: timing, type and situation of onset, provocation and palliation, quality, region and radiation, intensity (NRS), drug administration (type and dosage), and drug response.

Our antalgic approach included psychological therapy for the patient and his parents, ice application three times a day for 20 minutes, and pharmacological treatment.

Then, psychological therapy consisted of psychological interviews for the family and for the child and focused on the way pain changed family's structure and social life. Nonpharmacological pain management techniques (relaxation techniques such as breathing techniques and guided imagery) were taught to the child.

The pharmacological therapy consisted of gradual reduction of the steroid dosage, Lansoprazole 15 mg (1/day), Etoricoxib 30 mg 1/day, Paracetamol + Codeine at need, and acetylsalicylic acid suspension.

Furthermore, biochemical markers of bone turnover were measured.

25-Hydroxy-vitamin D and parathormone (PTH) levels were normal. Markers of bone formation (bone-specific alkaline phosphatase, osteocalcin, and N-terminal propeptide of type I procollagen) were found normally increased, according to the somatic growth related to age. Markers of bone resorption were found elevated as well, reflecting a huge bone resorption activity, related to the disease: urinary deoxypyridinoline (U-DPD) was 24,6 nmol/mmol; sieric carboxyterminal cross-linked telopeptide of type I collagen (S-ICTP) was 1549 pg/mL.

After steroid suspension, despite anti-inflammatory drugs use, pain immediately reappeared and the swelling worsened.

On the basis of clinical and biochemical data, zoledronic acid was administered intravenously (0,05 mg/kg in a single dose) in combination with 25-hydroxy-vitamin D (100.000 UI in a single dose before the infusion and then 400 UI daily) and calcium carbonate (1000 mg/daily). No side effects were reported. An improvement in pain and swelling was reported within few days and pain completely disappeared within 1 month from infusion. The patient was closely monitored and biochemical markers of bone resorption were measured two months after the zoledronic infusion: U-DPD and S-ICTP were decreased to 17,22 nmol/mmol and 1033 pg/mL.

After 8 months, pain appeared again and a second infusion of zoledronic acid was performed and no side effects were reported. We performed biochemical markers of bone resorption just before the second infusion of zoledronate (U-DPD was 33,2 nmol/mmol and S-ICTP was 2096 pg/mL) and two months after the second infusion, when U-DPD and S-ICTP concentrations were 22 nmol/mmol and 1362,4 pg/mL, respectively (Table 1).

**Figure 3:**
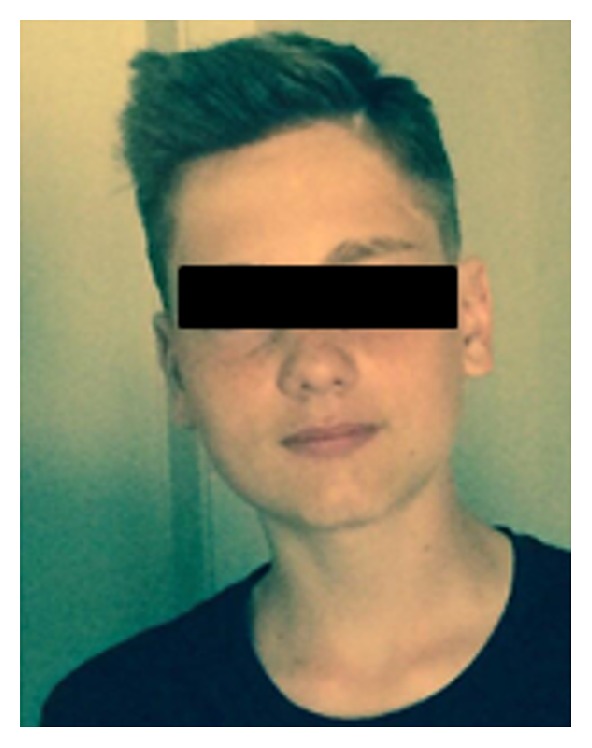
How our patient looks like now.

The patient is still in follow-up and refers no pain (Figure 3).

The results obtained thanks to ZOL infusion were considered satisfying by the patient and physicians. A previous surgery plan was cancelled and psychological support was no longer needed.

## 3. Discussion 

Fibrous dysplasia (FD) of bone is a rare skeletal disorder characterized by proliferation of fibrous tissue in bone marrow, often associated with bone pain, bone deformities, and fractures.

Affected bones are characterized by an enhanced osteoclastic activity; bone scans show lytic and cystic lesions with reduced cortex thickness and, sometimes, widening of the bone; biochemical markers of bone turnover can be elevated, especially in patients with large lesions.

The rationale for the use of antireabsorptive agents such as bisphosphonates in the treatment of FD is supported by histologic findings which often show high numbers of osteoclasts and evidence of bone resorption in FD lesions. Bisphosphonates improve the increased turnover of bone in FD by inhibiting osteoclast activity both directly and indirectly.

Bone pain is a common occurrence in FD and is often the presenting symptom; the pathophysiology of bone pain in this specific disorder is not well established.

Currently, the most commonly used analgesics to control FD pain are nonsteroidal anti-inflammatory drugs, bisphosphonates, and opiates.

Here, we present the case of an adolescent male with nociceptive persistent pain due to craniofacial fibrous dysplasia, successfully treated with zoledronic acid.

We observed a clear reduction of the biochemical markers of bone turnover, a prompt disappearance of pain, and a reduction of swelling after intravenous zoledronic acid.

Pain intensity was high and not responding to common analgesic drugs. It heavily interfered with daily activities forcing the child and his family to adjust their life to the disease and compromising their quality of life. For this reason, the psychologist of our team assessed this case and activated a psychologist support pathway.

Actually, no guidelines are available for the management of pain in FD.

Bisphosphonates are suggested as potential treatment since they inhibit osteoclastic bone resorption.

Some studies regarding the use of these drugs to reduce refractory pain in adults with FD are currently available. In their work, Boyce et al. [14] analyzed 24 adults and 16 children with polyostotic FD. Alendronate and placebo were administered over a 24-month period in 6-month cycles. Dosing was stratified by weight: 40 mg daily for subjects >50 kg, 20 mg for 30–50 kg, and 10 mg for 20–30 kg. Results of this study suggest that alendronate leads to a reduction in the bone resorption markers and improvement in bone mineral density (BMD) (*P* > 0.001) but has no significant effect on pain or functional parameters.

Zoledronic acid is a third-generation aminobisphosphonate and is currently used in adult patients for different bone diseases: this drug is a valid option in patients with metastasis of prostatic or breast cancer and in patients with Paget disease or osteoporosis [15–18].

Two case reports describe the wide efficacy of zoledronic acid for PFD in adults. Wu et al. [19] reported a case of PFD with advanced and extensive bone destruction in the skull and ribs. In this experience, ZOL was administered i.v. (5 mg) with a successful resolution of symptom and biochemical and radiological alterations [19]. Mansoori et al. [20] described the case of a 32-year-old man with PDF of the cranium treating with ZOL as second-line pharmacological treatment (the first drug administered was PAM). They reported a dramatic improvement of symptoms, of ALP levels, and of radiologic features thanks to ZOL [20]. In these works, no side effects were reported [19, 20].

These papers suggest that ZOL therapy is effective in PDF with severe bone destruction and is safe for long-term therapy in adults.

Only few studies about intravenous use of bisphosphonates in children with FD have been so far published. Kos et al. reported pain relief in 6 children affected by monostotic FD treated with intravenous pamidronate [21].

Zoledronic acid was used in a paediatric population in a randomized and controlled analysis, in which Barros et al. [10] compared the uses of zoledronic acid and pamidronate in 23 children affected by osteogenesis imperfecta. Results suggested that zoledronic acid is safe and effective in improving clinical and densitometric characteristics, similar to pamidronate [10].

A case of mandibular FD in an older girl was reported by Mäkitie et al. [22]; they described the history of a 12-year-old girl with pain, swelling, and disfigurement due to the growth of a lesion localized in the right side of the jaw. In this case, zoledronic acid (0,05 mg/kg) was used every six months but after a previous pamidronate infusion. Results were excellent with reduction of overall signs of disease [22].

Despite a significant number of papers available in literature about the use of pamidronate and alendronate in children affected by FD, no significant confirmations are present concerning administration of zoledronic acid for treatment of FD in the paediatric population (Table 2).

In our experience, we chose to use zoledronate because it has safety comparable to pamidronate in children [10] and can be safely infused over 15 minutes rather than the 2 hours recommended for pamidronate; moreover, it can be administered less frequently.

In this way, days of hospitalization of absence from school were minimized.

## 4. Conclusions

Our case report shows the safety of the use of zoledronic acid in an adolescent male and the efficacy for pain management in craniofacial fibrous dysplasia. Further research is needed to assess its safety and efficacy in paediatric population.

## Figures and Tables

**Table 1:** Markers of bone resorption after and before zoledronic i.v. administrations.

	Urinary deoxypyridinoline (U-DPD) nmol/mmol	Sieric carboxyterminal cross-linked telopeptide of type I collagen (S-ICTP) pg/mL
Before the first zoledronic infusion	24,6	1549
After the first zoledronic infusion	17,22	1033
Before the second zoledronic infusion	33,2	2096
After the second zoledronic infusion	22	1362,4

**Table 2:** Case reports about use of bisphosphonates in paediatric fibrous dysplasia.

Source	Subjects	Disease	Signs and symptoms	Treatment	Efficacy of treatment
Hart et al. [4]	Male with cherubism diagnosed when he was 6 years old	Cherubism	Pain localized in mandible	Calcitonin and bisphosphonates	Pain reduction

Zumkeller et al. [5]	Male child 8 years old	McCune-Albright syndrome and pituitary adenoma	Polyostotic dysplasia, Cafè-au-lait spots, and precocious puberty	Pamidronate and sandostatin	Increased mobility

Rastogi et al. [6]	15-year-old female	McCune-Albright syndrome	Recurrent fractures of femur by minor trauma and chronic bone pain (right thigh)	Pamidronate i.v. (1 mg/kg/day for 3 days once in 3 months for a period of 1 year), 25-hydroxy-vitamin D, and calcium	Pain reduction and no fractures developed over three years of follow-up

Kochar and Kulkarni [7]	2- and 8-year-old female	McCune-Albright syndrome	Generalized bone pain, recurrent fractures by minor trauma, fatigue, and bone deformities	Pamidronate i.v.	Remarkable clinical improvement

Atsali et al. [8]	14-year-old female	McCune-Albright syndrome	Cystic lesions of the right humerus incidentally discovered, bone pain, and a history of fracture of the distal humerus by minor trauma	At first, pamidronate i.v. and then i.v. zoledronic acid (5 mg annually)	During treatment, pain reduction, no fractures, and increased BMD after 4 years of therapy

Bieniasz et al. [9]	12-year-old female	Craniofacial FD	Orbital cavity and sinuses lesions	Pamidronate	Clinical improvement

Mäkitie et al. [22]	12-year-old female	Craniofacial FD	Mandibular lesions, swelling, disfigurement, and pain of the right side of the jaw	Pamidronate i.v. (1 mg/kg on 3 consecutive days at 4-month intervals), in the following year, the therapy was switched to zoledronic acid i.v. (0.05 mg/kg once every 6 months)	Pain and disfigurement reduction

Khadilkar et al. [11]	3-year-old male	Polyostotic FD	Recurrent fractures of the left femur, pain, and asymmetry	Oral alendronate	Pain reduction and no fractures

Chattopadhyay et al. [12]	17-year-old female	Polyostotic FD and hypophosphatemia	Swelling of the left forearm, progressive bowing deformity of the lower limb, widening of the wrist, decrease in linear growth with sever short stature, rickets, and osteomalacia	Oral alendronate, phosphate, and 25-hydroxy-vitamin D	Clinical improvement

Jayaraman et al. [13]	18-year-old female	Polyostotic FD	Pain, swelling and fracture in the right middle finger after minor trauma, and expansive lesions in the right hand and in the superior pubic ramus	Oral alendronate (70 mg weekly), calcium carbonate 1000 mg daily, and 25-hydroxy-vitamin D 400 IU daily	Radiographic improvement of bone lesions
